# mTORC2 regulates lipid metabolism-driven TAMs via the PPAR-γ/CD36 pathway to promote liposarcoma progression

**DOI:** 10.1080/21623945.2026.2665903

**Published:** 2026-04-29

**Authors:** Weihua Xiao, Jingdan Sheng, Chunjiao Liu, Junqiang Li, Peng Shu, Maofen Jiang, Min Wang, Ji He, Haifen Ma, Yaxin Ding

**Affiliations:** Department of Pathology, Beilun People’s Hospital, Ningbo, Zhejiang, P. R. China

**Keywords:** mTORC2, tumour-associated macrophages, liposarcoma, fatty acid metabolism, PPAR-γ/CD36 pathway

## Abstract

Tumour-associated macrophages (TAMs) exert a pivotal function in tumour progression, and M2-type TAMs are closely linked to tumour-promoting functions. Mechanistic target of rapamycin complex 2 (mTORC2) may mediate TAM polarization and subsequent tumour development, yet their roles in liposarcoma (LPS) remain unclear. Macrophage polarization was assessed via flow cytometry for CD206. Western blot for Arg-1, Rictor, PPAR-γ, CD36, ACSL1 and CPT2, qPCR for IL-10, Arg-1 and Ym1, and ELISA for IL-10 secretion. Fatty acid metabolism was evaluated using free fatty acid (FFA) uptake assays and Oil Red O staining for intracellular lipid droplets. A Transwell assay was established to assess the effect of treated macrophages on LPS cell biology, and EdU assays were used for proliferation assessment. Transwell migration and invasion assays were utilized for assessing cellular motility. IL-4 induced RAW264.7 macrophages to undergo M2 polarization, characterized by upregulated CD206, Arg-1, and IL-10. During this process, mTORC2 was activated, promoting FFA uptake, lipid droplet accumulation, and fatty acid oxidation via the PPAR-γ/CD36 axis. Co-culture experiments showed that IL-4-polarized M2 macrophages enhanced LPS cell proliferation, migration, and invasion; these effects were inhibited by JR-AB2-011 and restored by LPA, confirming mTORC2-PPAR-γ/CD36-mediated TAMs drive LPS progression. mTORC2 regulates M2 TAM polarization and metabolic reprogramming via the PPAR-γ/CD36 pathway, thereby promoting LPS cell proliferation, migration, and invasion.

## 1. Introduction

Liposarcoma (LPS) originates from adipocyte-derived malignant cells. Clinically, it has become a major clinical challenge due to its characteristic extensive growth before detection and the lack of substantially novel and effective therapeutic strategies [1]. Despite advances in surgical resection, chemotherapy, radiotherapy and immunotherapy, the prognostic outlook for patients advanced or recurrent LPS remains suboptimal [2], underscoring the urgent need to develop innovative therapeutic approaches.

Among the heterogeneous cellular constituents of the tumour microenvironment (TME), tumour-associated macrophages (TAMs) serve as core regulators of tumour biology [3]. Studies have shown that TAMs exhibit significant functional plasticity and can differentiate into distinct phenotypes in response to signals from the TME, their heterogeneity is crucial to their role in tumour development [4]. Classically activated M1 macrophages exert antitumor effects via the secretion of pro-inflammatory cytokines and mediating tumour cell phagocytosis, while alternatively activated M2 macrophages – the dominant phenotype of TAMs in most tumours – promote tumour progression through immune suppression, tissue repair, and supporting tumour cell proliferation [5,6]. Research has confirmed that TAMs in LPS predominantly exhibit an M2-like phenotype, and contribute to driving the malignant progression of the disease [7,8]. Extensive studies have found that the functional phenotype of TAMs is closely correlated with their metabolic reprogramming [9,10]. Unlike M1 macrophages, which predominantly rely on glycolysis for energy supply [11], M2 macrophages exhibit a significantly enhanced dependence on oxidative phosphorylation [12], a process induced by the upregulation of fatty acid oxidation (FAO) [13]. The unique lipid metabolism pattern of TAMs provides support for this metabolic shift [14]. Importantly, studies have verified that the intracellular lipid droplets modulate polarization of macrophages phenotype [15]. Targeting the process of lipid droplet formation in TAMs can reverse their immune-suppressive properties, underscoring the core role of lipid metabolism in sustaining the pro-tumour function of TAMs [14]. This association between TAMs lipid metabolism and LPS progression suggests that regulating the metabolic reprogramming of TAMs may be a viable therapeutic target.

The mechanistic target of rapamycin (mTOR) is an evolutionarily conserved serine/threonine kinase belonging to the phosphatidylinositol 3-kinase-related kinase (PIKK) family [16], and it plays a central role in regulating macrophage polarization [17]. mTOR serves as the catalytic subunit of two distinct complexes, mTOR complex 1 (mTORC1) and mTOR complex 2 (mTORC2), which differ in their component composition and biological functions in macrophages [18]. Both complexes are involved in the M2 polarization process, but their roles differ [19,20]. Studies have demonstrated that M2-polarizing cytokines can enhance the activation of mTORC1, thereby participating in the metabolic and functional reprogramming of M2 macrophages [21]. In contrast, mTORC2 is an essential factor for M2 polarization of macrophages [22]. Studies have further shown that mTORC2 is involved in regulating the lipid metabolism of M2 macrophages [23]. Crucially, mTORC2 regulates lipid metabolism and M2 polarization through the peroxisome proliferator-activated receptor γ (PPAR-γ) [24]. Notably, PPAR-γ is a key transcription factor involved in lipid metabolism and is essential for the pro-tumour polarization of TAMs [25]. This suggests that mTORC2 can act as an upstream regulator of the PPAR-γ, linking signalling pathways and metabolic reprogramming in TAMs.

In this present, we found that during M2 polarization of macrophages induced by IL-4, mTORC2 promotes fatty acid uptake and lipid droplet accumulation via the PPAR-γ pathway, thereby enhancing FAO and maintaining the M2-like pro-tumour phenotype of TAMs. These TAMs subsequently promote the biological behaviours of LPS cells, ultimately driving the progression of LPS. This study provides a new insights for investigating the interaction between immunity and metabolism in LPS.

## 2. Methods

### 2.1. Cell culture

Human liposarcoma cell lines SW872 and 94T778, as well as the murine macrophage cell line RAW264.7, were purchased from the American Type Culture Collection (ATCC, USA). These cells were cultured in Dulbecco’s Modified Eagle’s Medium (DMEM, Gibco, USA) supplemented with 10% (v/v) foetal bovine serum (FBS, Gibco) and 1% (v/v) penicillin-streptomycin solution. The culture environment was maintained at 37°C in a humidified atmosphere with 5% CO_2_.

### 2.2. Macrophage polarization induction model

RAW264.7 cells were seeded in 6-well plates, they were treated with 50 ng/mL interleukin-4 (IL-4, BD Biosciences, USA) to induce M2 polarization [19]. Cells were pre-treated with 1 μM JR-AB2-011 (mTORC2 inhibitor, Selleck Chemicals, USA) [19] for 1 h prior to IL-4 stimulation [19]. 10 μM lysophosphatidic acid [26] (LPA, PPAR-γ activator, Sigma-Aldrich, USA), was added simultaneously with IL-4 in the IL-4+JR-AB2-011+LPA group. Control groups received an equal volume of vehicle (PBS or DMSO, Sigma-Aldrich) without any treatment.

### 2.3. Transwell co-culture experiments

RAW264.7 cells were treated as described above for 48 h, then harvested and seeded into the upper chamber of Transwell inserts (0.4 μm pore size, Corning, USA) at 1 × 10^4^ cells/insert. SW872 or 94T778 cells were seeded in the lower chamber of 24-well plates at 5 × 10^4^ cells/well [27]. Co-cultures were maintained for 48 h before subsequent assays.

### 2.4. Flow cytometry

After 48 hours of experimental treatment, RAW264.7 cells were collected and resuspended in FACS buffer at a final concentration of 1 × 106 cells/ml. Subsequently, the cells were incubated with anti-mouse CD206 monoclonal antibody (1:100, BD Biosciences, USA) under dark conditions for 30 minutes. Then, the cells were washed with FACS buffer to remove the unbound primary antibody, and subsequently analysed using the BD FACSCanto II flow cytometer (BD Biosciences, USA). The data were analysed using FlowJo software (version 10.8), and the proportion of CD206-positive cells in each experimental group was determined.

### 2.5. ELISA assay

After 48 hours of experimental treatment, the culture supernatant of RAW264.7 cells was collected and centrifuged at 1200×g for 10 minutes to precipitate cell debris. The IL-10 level in the clarified supernatant was determined using a commercial mouse IL-10 ELISA kit (E-EL-M0046, Elabscience, China) according to the manufacturer’s recommended procedure. The absorbance was measured using a microplate reader (Bio-Rad) at 450 nanometres, and the IL-10 concentration of each sample was calculated by interpolating the absorbance value with the standard curve.

### 2.6. EdU staining for cell proliferation

SW872 or 94T778 cells were placed in a co-culture system and treated with 10 μM EdU (Thermo Fisher Scientific) for 2 hours at 37°C. Subsequently, the cells were fixed with 4% paraformaldehyde for 15 minutes, followed by permeabilization with 0.5% Triton X-100 for 15 minutes. Cell nuclei were stained with DAPI (Thermo Fisher Scientific). Images were captured using a fluorescence microscope (Olympus, Japan).

### 2.7. Transwell assays

After completion of the co-culture period, SW872 and 94T778 cells were harvested and plated into the upper chambers of Transwell inserts (Corning). The lower layer were filled with DMEM containing 20% foetal bovine serum as a chemical stimulus to drive cell directional migration. After incubation at 37°C for 24 hours, the cells that passed through the membrane and adhered to the lower surface of the chamber were fixed with 4% paraformaldehyde for 15 minutes, and then stained with 0.1% crystal violet (Sigma-Aldrich) for 20 minutes. Images were captured using an Olympus optical microscope, and the number of migrating cells was quantified by counting five random fields in each well. For the invasion experiment, the Transwell chamber was pre-coated with 50 μl of Matrigel (Corning). Subsequent steps were identical to the migration assay, except that the incubation time was extended to 48 h. Invaded cells were counted and analysed as described above.

### 2.8. Free fatty acid (FFA) uptake assay

FFA uptake was detected using the Free Fatty Acid Uptake Assay Kit (E-BC-K792-M, Elabscience) following the manufacturer’s protocol. RAW264.7 cells were seeded in 96-well plates at 1 × 10^4^ cells/well and treated for 48 h. Cells were incubated with FFA working solution for 30 min at 37°C, and 100 μL of detection buffer was added to each well. The fluorescence intensity was measured using a microplate reader, with the excitation wavelength set at 485 nm and the emission wavelength at 520 nm (Bio-Rad).

### 2.9. Oil Red O staining

RAW264.7 cells were seeded at 24-well plates and cultured with designated treatments for 48 h. For lipid staining, the cells were incubated together with the Oil Red O working solution (3:2 dilution of 0.5% Oil Red O-isopropanol stock with distilled water; Sigma-Aldrich) at room temperature. Non-specific dye binding was removed by rinsing thoroughly with distilled water, and nuclear counterstaining was conducted with haematoxylin (Sigma-Aldrich) for 2 min. Images were obtained using an Olympus light microscope equipped with a digital imaging system.

### 2.10. Western Blot

Total protein was extracted from RAW264.7 cells using RIPA lysis buffer (Beyotime, China). Equal amounts of protein were separated by SDS-PAGE, then transferred to PVDF membranes (Millipore, USA), and incubated with primary antibodies overnight at 4°C. Primary antibodies included anti-Rictor (1:1000), anti-Arg-1 (1:1000), anti-CD206 (1:1000), anti-CPT2 (1:1000), anti-ACSL1 (1:1000), anti-PPAR-γ (1:1000), anti-CD36 (1:1000), and anti-β-actin (1:5000), all from Cell Signaling Technology. Protein bands were visualized using an ECL Detection Kit (Millipore).

### 2.11. Quantitative polymerase chain reaction (qPCR)

Total RNA was extracted from RAW264.7 cells using TRIzol reagent (Thermo Fisher Scientific) per the manufacturer’s standard operating instructions. cDNA was synthesized from 1 μg of total RNA using the PrimeScript RT Reagent Kit with gDNA Eraser (Vazyme, China). Quantitative real-time PCR was performed on a StepOnePlus Real-Time PCR System (Thermo Fisher Scientific) using the SYBR Premix Ex Taq II Kit (Vazyme) to detect target gene amplification. The specific primer sequences for all target and reference genes are provided in Table 1. Relative mRNA expression levels were determined by the 2^−ΔΔCt^ method, with GAPDH used as the internal control for data normalization.

**Table 1.** Primer sequences.

Gene	Forward primer	Reverse primer
IL-10	CGGGAAGACAATAACTGCACCC	CGGTTAGCAGTATGTTGTCCAGC
Arg-1	GTGTACATTGGCTTGCGAGA	GGTCTCTTCCATCACCTTGC
Ym1	CAGTGTTCTGGTGAAGGAAATG	ACCCAGACTTGATTACGTCAAT
GAPDH	GGCAAGTTCAACGGCACAG	GCCAGTAGACTCCACGACAT

### 2.12. Statistical analysis

All data were presented as the mean ± SD using GraphPad Prism 10. Differences between groups were analysed using one-way ANOVA followed by Tukey’s post-hoc test. *p* < 0.05 was considered statistically significant.

## 3. Results

### 3.1. IL-4-induced M2-type macrophages promote the progression of LPS

**Figure 1.**
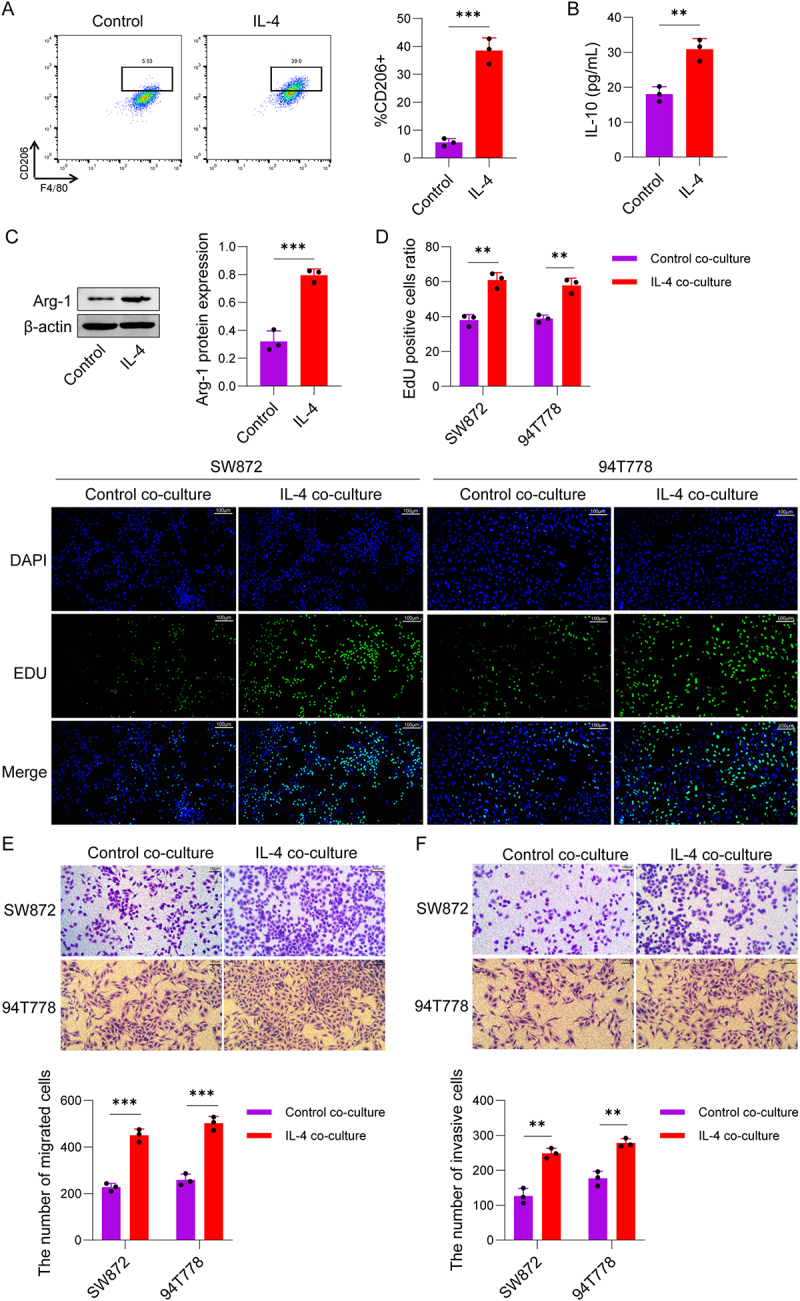
IL-4-Induced M2 macrophage polarization promotes the progression of LPS. RAW264.7 cells were divided into two groups: control group, IL-4 group. (A) Flow cytometry was used to detect the expression level of CD206 on RAW264.7 cells in each group. (B) ELISA was performed to measure the secretion level of IL-10 in RAW264.7 cells from each group. (C) Western Blot was employed to determine the protein expression level of Arg-1 in RAW264.7 cells of each group. A Transwell co-culture system was utilized, which included two groups: control co-culture group (LPS cells co-cultured with untreated RAW264.7 cells). IL-4 co-culture group (LPS cells co-cultured with RAW264.7 cells pre-treated with IL-4). (D) EdU incorporation assay was used to detect the proliferative activity of SW872 cells and 94T778 cells in each group. (E-F) Transwell migration and invasion assays were performed to evaluate the migration and invasion abilities of SW872 cells and 94T778 cells in each group. Data are presented as the mean ± SD. *p < 0.05, **p < 0.01, ***p < 0.001. Experiments were repeated independently at least three times.

To investigate whether IL-4 can induce the polarization of macrophages into the M2 phenotype, and hence regulate the progression of LPS-induced inflammatory responses processes. The results of flow cytometry (Figure 1(A)) indicated that the proportion of CD206-positive RAW264.7 cells was significantly elevated in the IL-4 group. ELISA results showed that the content of IL-10 were markedly higher in the IL-4 group (Figure 1(B)), which further confirmed that IL-4 could induce the polarization of macrophages into the M2 phenotype. The results further found that the relative expression level of Arg-1 protein in RAW264.7 cells was upregulated in the IL-4 group by Western Blot (Figure 1(C)). Notably, EdU proliferation assay results demonstrated that the proportion of EdU-positive cells was elevated in the IL-4 co-culture group (Figure 1(D)). Transwell migration assay results (Figure 1(E)) showed that the number of migrated SW872 and 94T778 cells was dramatically elevated in the IL-4 co-culture group, suggesting that M2-type macrophages could significantly promote the migration ability of LPS cells. Similarly, Transwell invasion assay results demonstrated a significant higher in the IL-4 co-culture group (Figure 1(F)). Collectively, these findings indicate that IL-4 can drive the biological behaviours of LPS cells via inducing RAW264.7 macrophages to polarize into the M2 phenotype, thereby facilitating the advancement of liposarcoma.

### 3.2. During M2 polarization, mTORC2 promotes lipid droplet accumulation and fatty acid oxidation

**Figure 2.**
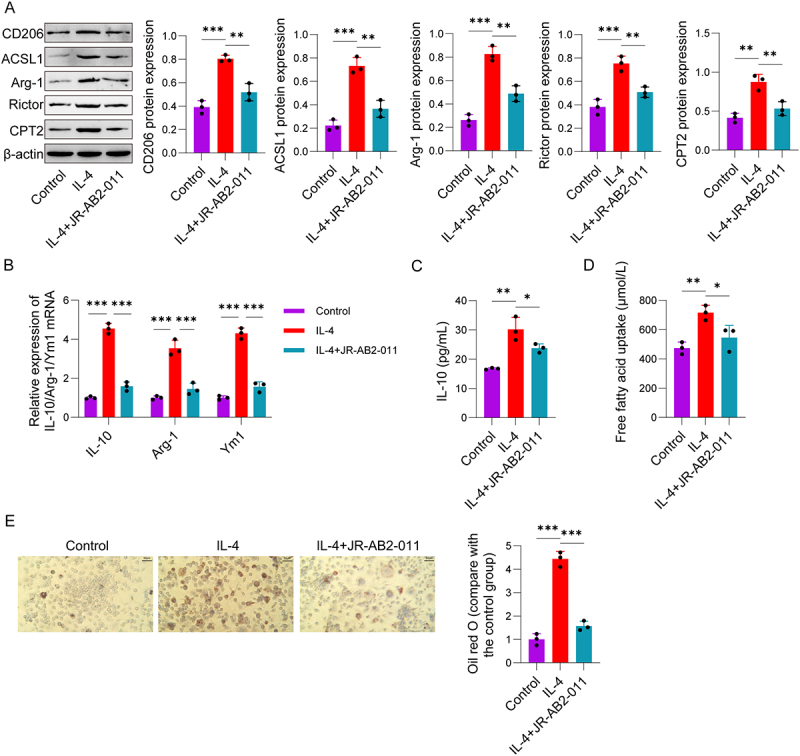
mTORC2 promotes lipid droplet accumulation and fatty acid oxidation during M2 polarization. RAW264.7 cells were divided into three groups based on treatment: control group, IL-4 group, IL-4+JR-AB2-011 group. (A) Western Blot was performed to detect the protein expression levels of Rictor, Arg-1, CD206, CPT2, and ACSL1. (B) qPCR was used to determine the mRNA expression levels of IL-10, Arg-1, and Ym1. (C) ELISA was conducted to measure the secretion of IL-10 in RAW264.7 cells. (D) The FFA uptake in RAW264.7 cells was detected. (E) Oil Red O staining was used to determine the intracellular lipid droplet content in RAW264.7 cells. Data are presented as the mean ± SD. *p < 0.05, **p < 0.01, ***p < 0.001. Experiments were repeated independently at least three times.

mTORC2 can regulate the process of fatty acid oxidation by modulating the PPAR-α signalling pathway [28], and the metabolic reprogramming dependent on fatty acid oxidation in M2-type TAMs is a key mechanism underlying their role in promoting tumour metastasis [29]. Western Blot results demonstrated that of Rictor, Arg-1, CD206, CPT2, and ACSL1 protein expression levels were notably elevated in the IL-4 group; conversely, treatment with JR-AB2-011 (IL-4+JR-AB2-011 group) led to a reduction in the expression of these proteins (Figure 2(A)). Meanwhile, qPCR assays found that the mRNA levels of *IL-10*, *Arg-1*, and *Ym1* were higher in the IL-4 group, whereas these transcript levels were notably decreased in the IL-4+JR-AB2-011 group. These further confirm that mTORC2 is involved in regulating IL-4-induced M2 polarization. ELISA results (Figure 2(C)) demonstrated that the concentration of IL-10 in the cell culture supernatant of the IL-4 group was elevated. However, the secretion of IL-10 was significantly reduced in the IL-4+JR-AB2-011 group. These results found that the activation of mTORC2 is essential for IL-4-induced IL-10 secretion, which is a key functional characteristic of M2 macrophages. Further FFA uptake assay results (Figure 2(D)) showed that the relative FFA uptake level was dramatically increased in the IL-4 group, while the FFA uptake level was significantly decreased in the IL-4+JR-AB2-011 group. Consistently, Oil Red O staining results (Figure 2(E)) indicated that the intracellular lipid droplet content was significantly increased in the IL-4 group, whereas JR-AB2-011 treatment could significantly reduce lipid droplet accumulation. These findings suggest that during IL-4-induced M2 macrophage polarization, mTORC2 enhances intracellular lipid droplet accumulation and fatty acid oxidation. This mTORC2-mediated reprogramming of fatty acid metabolism may play a key function in maintaining the phenotypic and functional characteristics of M2 macrophages.

### 3.3. mTORC2 promotes intracellular lipid droplet accumulation and fatty acid oxidation in macrophages via the PPAR-γ/CD36 axis

**Figure 3.**
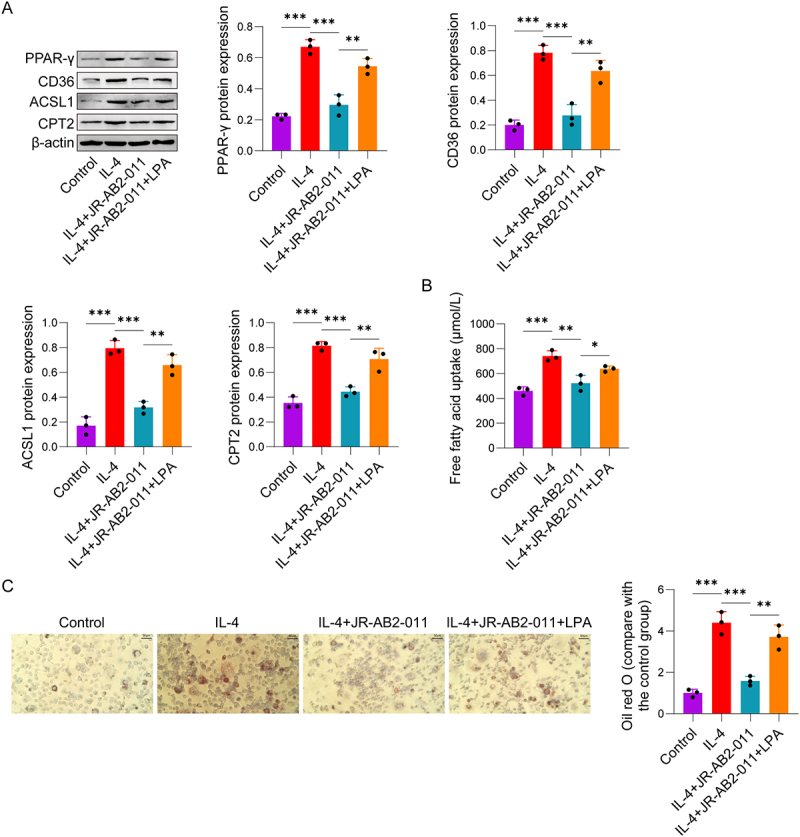
mTORC2 regulates lipid metabolism in macrophages via the PPAR-γ/CD36 axis. RAW264.7 cells were divided into four groups according to the treatment strategy: control group, IL-4 group, IL-4+JR-AB2-011 group, IL-4+JR-AB2-011+LPA group. (A) Western Blot was performed to detect the protein expression levels of PPAR-γ, CD36, ACSL1, and CPT2 in RAW264.7 cells. (B) The FFA uptake in RAW264.7 cells of each group was detected. (C) Oil Red O staining was used to determine the intracellular lipid droplet content in RAW264.7 cells of each group. Data are presented as the mean±SD. *p < 0.05, **p < 0.01, ***p < 0.001. Experiments were repeated independently at least three times.

mTORC2 regulates lipid synthesis by modulating the expression of adipogenic genes through PPARγ [30]. To further identify the downstream mediator of mTORC2 in this process, we focused on the PPAR-γ, a well-characterized pathway regulating lipid metabolism and M2 polarization [31,32]. The results found that the PPAR-γ, CD36, ACSL1, and CPT2 protein expression levels were significantly increased in the IL-4 group by Western Blot. However, the expression of these proteins was significantly decreased in the IL-4+JR-AB2-011 group, while the expression levels of PPAR-γ, CD36, ACSL1, and CPT2 were significantly elevated in the IL-4+JR-AB2-011+LPA group (Figure 3(A)). The results of FFA uptake assay exhibited a consistent trend with the protein expression patterns (Figure 3(B)). Further Oil Red O staining results found that the intracellular lipid droplet content was significantly increased in the IL-4 group. In contrast, lipid droplet accumulation was markedly reduced in the IL-4+JR-AB2-011 group, while treatment with LPA significantly reversed the inhibitory effect of JR-AB2-011 (Figure 3(C)). These findings indicate that mTORC2 regulates PPAR-γ/CD36 to enhance FFA uptake, as well as the subsequent lipid droplet accumulation and fatty acid oxidation.

### 3.4. TAMs regulated by the mTORC2/PPAR-γ/CD36 pathway promote the progression of LPS

**Figure 4.**
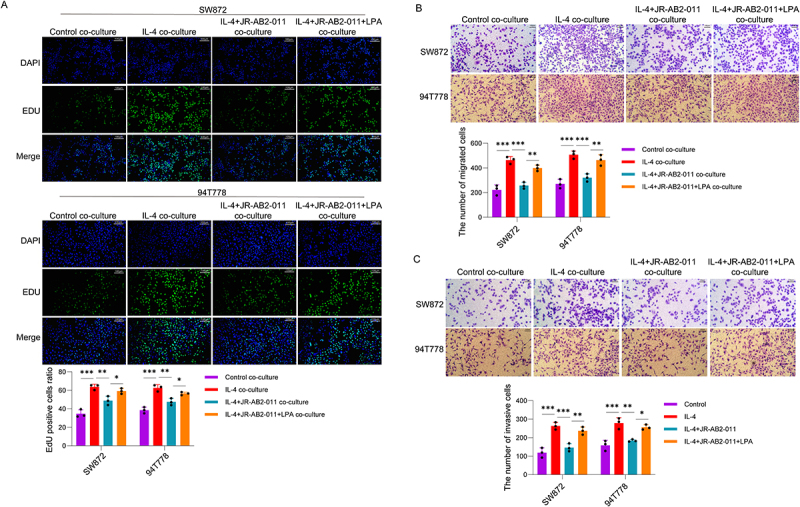
mTORC2 regulates TAM via PPAR-γ/CD36 pathway to promote LPS progression. LPS cells (SW872 and 94T778) were co-cultured with RAW264.7 macrophages, and the groups were divided as follows: control co-culture group, IL-4 co-culture group, IL-4+JR-AB2-011 co-culture group, IL-4+JR-AB2-011+LPA co-culture group. (A) EdU assay was used to detect the proliferation of SW872 and 94T778 cells in each co-culture group. (B-C) Transwell migration and invasion assays were performed to evaluate the migration and invasion abilities of SW872 and 94T778 cells in each co-culture group. Data are presented as the mean±SD. *p < 0.05, **p < 0.01, ***p < 0.001. Experiments were repeated independently at least three times.

The induction of PPAR-γ/CD36 pathway-dependent fatty acid oxidation promotes the polarization state of tumour-associated macrophages [33]. To explore the regulatory function of the mTORC2 signalling pathway in M2 polarization induced by IL-4 during the regulation of LPS cell biological behaviours via the PPAR-γ/CD36 axis. The EdU assay results (Figure 4(A)) indicated that the proliferation rate was upregulated in the IL-4 co-culture group. The proliferation rate was significantly decreased in the IL-4+JR-AB2-011 co-culture group. Notably, the proliferation rate was significantly higher in the IL-4+JR-AB2-011+LPA co-culture group than in the IL-4+JR-AB2-011 co-culture group. The Transwell migration assay results (Figure 4(B)) showed that the number of migrated SW872 and 94T778 cells was increased in the IL-4 co-culture group. The number of migrated cells was decreased in the IL-4+JR-AB2-011 co-culture group. However, the number of migrated cells was significantly reversed in the IL-4+JR-AB2-011+LPA co-culture group. The invasion results accorded with the migration results (Figure 4(C)). These results indicate that mTORC2 regulates the formation of tumour-associated macrophages (TAMs) via the PPAR-γ/CD36 pathway, and ultimately facilitating the progression of liposarcoma.

## 4. Discussion

TAMs, as a key component of the TME, have been increasingly recognized as a driver of malignant progression in different tumours [34]. In this present, we investigated the role of mechanistic target of mTORC2 in regulating TAM biology and LPS progression.

The functional plasticity of macrophages allows them to switch between M1 and M2 phenotypes in response to TME cues, and this polarization process is tightly associated with tumour outcomes [34]. Previous studies have reported that M2-type TAM infiltration correlates with poor prognosis in LPS [35]. Our results confirmed that IL-4 effectively induces RAW264.7 macrophages to polarize into M2-type cells. More importantly, we demonstrated that these IL-4-polarized M2 macrophages significantly enhance malignant biological behaviours in LPS, highlighting the critical role of M2 TAMs in LPS cell malignant progression, supporting the notion that targeting M2 macrophages could disrupt the pro-tumour TME in LPS.

The mTOR signalling pathway has emerged as a central regulator of macrophage polarization, with mTORC1 and mTORC2 playing distinct roles [36]. While mTORC1 is primarily involved in glycolytic metabolism of M1 macrophages [37], recent evidence suggests that mTORC2 is critical for M2 polarization and lipid metabolism [38]. Our study further showed that inhibition of mTORC2 downregulates M2 markers and FAO-related enzymes and reduces IL-10 secretion. Concurrently, the results revealed that inhibition of mTORC2 partially attenuates IL-4-induced increases in FFA uptake and intracellular lipid droplet accumulation – key features of M2 macrophage metabolic reprogramming [39]. This study confirmed that mTORC2 is not only required for M2 phenotypic polarization but also for the metabolic shift towards FAO, which is known to sustain M2 functional properties.

Previous studies found that mTORC2 regulates lipid metabolism via PPAR-γ^[31]^, but we extend this mechanism to LPS and demonstrate its functional relevance to tumour progression. Our results showed that inhibition of mTORC2 make macrophages lose their ability to promote LPS cell proliferation, migration, and invasion, while PPAR agonist restores this pro-tumour effect. This confirms that the mTORC2/PPAR-γ/CD36 axis is not only a regulator of TAM metabolism but also a driver of LPS malignant behaviours, bridging TAM metabolic reprogramming to tumour progression.

In conclusion, our study uncovers a novel mechanism by which mTORC2 regulates lipid metabolism-driven TAMs via the PPAR-γ/CD36 pathway to promote LPS progression. Unlike previous studies that focused on glycolysis in LPS cells, we shift the focus to TAM lipid metabolism, providing a new perspective on LPS progression. Notably, it identifies the mTORC2/PPAR-γ/CD36 axis as a potential therapeutic target for LPS.

Despite these strengths, this study has several limitations that should be addressed in future research. Our experiments were conducted using cell lines, which may not fully recapitulate the complexity of the in vivo TME. Future studies using LPS patient-derived xenografts or genetically engineered mouse models will be critical to validate our findings *in vivo*. In addition, we did not explore the clinical relevance of the mTORC2/PPAR-γ/CD36 axis in LPS patients, future studies analysing patient samples and their correlation with prognosis will help translate our findings into clinical practice in LPS.

## 5. Conclusions

In conclusion, our study demonstrates that mTORC2-mediated metabolic reprogramming of TAMs via the PPAR-γ/CD36 axis is a key driver of LPS progression. Targeting this pathway holds great promise as a novel therapeutic strategy for LPS.

## Data Availability

The datasets cannot be shared due to privacy concerns, but available from the corresponding author on reasonable request.

## Abbreviation

mTORC2: Mechanistic Target of Rapamycin Complex 2;
TAMs: Tumour-Associated Macrophages;
LPS: Liposarcoma;
FAO: Fatty Acid Oxidation;
PPAR-γ: Peroxisome Proliferator-Activated Receptor;
TME: Tumour Microenvironment;
FFA: Free Fatty Acid.

## Highlights

1. M2-type tumor-associated macrophages significantly promote LPS progression.
2. mTORC2 regulates the lipid metabolism and pro-tumour function of TAMs through the PPAR-γ/CD36 axis.
3. mTORC2/PPAR-γ/CD36 axis may serve as a potential therapeutic target for LPS.

## References

[1] Liu H, Wang X, Liu L, et al. Targeting liposarcoma: unveiling molecular pathways and therapeutic opportunities. Front Oncol. 2024;14:1484027. doi: 10.3389/fonc.2024.1484027 39723387 PMC11668776

[2] Zhou XP, Xing JP, Sun LB, et al. Molecular characteristics and systemic treatment options of liposarcoma: a systematic review. Biomed Pharmacother = Biomedecine Pharmacotherapie. 2024;178:117204. doi: 10.1016/j.biopha.2024.117204 39067161

[3] Yang Y, Li S, To KKW, et al. Tumor-associated macrophages remodel the suppressive tumor immune microenvironment and targeted therapy for immunotherapy. J Exp Clin Cancer Res. 2025;44(1):145. doi: 10.1186/s13046-025-03377-9 40380196 PMC12083052

[4] Gao J, Liang Y, Wang L. Shaping polarization of tumor-associated macrophages in cancer immunotherapy. Front Immunol. 2022;13:888713. doi: 10.3389/fimmu.2022.888713 35844605 PMC9280632

[5] Shao N, Qiu H, Liu J, et al. Targeting lipid metabolism of macrophages: a new strategy for tumor therapy. J Adv Res. 2025;68:99–13. doi: 10.1016/j.jare.2024.02.009 38373649 PMC11785569

[6] Boutilier AJ, Elsawa SF. Macrophage polarization states in the tumor microenvironment. Int J Mol Sci. 2021;22(13):6995. doi: 10.3390/ijms22136995 34209703 PMC8268869

[7] Nabeshima A, Matsumoto Y, Fukushi J, et al. Tumour-associated macrophages correlate with poor prognosis in myxoid liposarcoma and promote cell motility and invasion via the HB-EGF-EGFR-PI3K/Akt pathways. Br J Cancer. 2015;112(3):547–555. doi: 10.1038/bjc.2014.637 25562433 PMC4453656

[8] Schroeder BA, Lafranzo NA, Lafleur BJ, et al. CD4+ T cell and M2 macrophage infiltration predict dedifferentiated liposarcoma patient outcomes. J Immunother Cancer. 2021;9(8):e002812. doi: 10.1136/jitc-2021-002812 34465597 PMC8413967

[9] Chen Y, Wu G, Li M, et al. LDHA-mediated metabolic reprogramming promoted cardiomyocyte proliferation by alleviating ROS and inducing M2 macrophage polarization. Redox Biol. 2022;56:102446. doi: 10.1016/j.redox.2022.102446 36057161 PMC9437906

[10] Zhong X, Gong S, Meng L, et al. Cordycepin modulates microglial M2 polarization coupled with mitochondrial metabolic reprogramming by targeting HKII and PDK2. Adv Sci (Weinheim, Baden-Wurttemberg, Ger). 2024;11(31):e2304687. doi: 10.1002/advs.202304687 PMC11336950 38889331

[11] Soto-Heredero G, Gómez DE Las Heras MM, Gabandé-Rodríguez E, et al. Glycolysis - a key player in the inflammatory response. FEBS J. 2020;287(16):3350–3369. doi: 10.1111/febs.15327 32255251 PMC7496292

[12] Zhao SJ, Kong FQ, Jie J, et al. Macrophage MSR1 promotes BMSC osteogenic differentiation and M2-like polarization by activating PI3K/AKT/GSK3β/β-catenin pathway. Theranostics. 2020;10(1):17–35. doi: 10.7150/thno.36930 31903103 PMC6929615

[13] Ahn S, Park JH, Grimm SL, et al. Metabolomic rewiring promotes endocrine therapy resistance in breast cancer. Cancer Res. 2024;84(2):291–304. doi: 10.1158/0008-5472.CAN-23-0184 37906431 PMC10842725

[14] Feng X, Zhang J, Yu B, et al. Lipid metabolic reprogramming in tumor-associated macrophages: a key driver of functional polarization and tumor immunomodulation. Crit Rev Oncol Hematol. 2025;215:104881. doi: 10.1016/j.critrevonc.2025.104881 40783075

[15] Cabodevilla AG, Son N, Goldberg IJ. Intracellular lipase and regulation of the lipid droplet. Curr Opin Lipidol. 2024;35(2):85–92. doi: 10.1097/MOL.0000000000000918 38447014 PMC10919935

[16] Mao B, Zhang Q, Ma L, et al. Overview of research into mTOR inhibitors. Molecules. 2022;27(16):5295. doi: 10.3390/molecules27165295 36014530 PMC9413691

[17] Zhang J, Hao L, Li S, et al. mTOR/HIF-1α pathway-mediated glucose reprogramming and macrophage polarization by Sini decoction plus ginseng soup in ALF. Phytomedicine. 2025;137:156374. doi: 10.1016/j.phymed.2025.156374 39798342

[18] Zhang X, Evans TD, Chen S, et al. Loss of macrophage mTORC2 drives atherosclerosis via FoxO1 and IL-1β signaling. Circ Res. 2023;133(3):200–219. doi: 10.1161/CIRCRESAHA.122.321542 37350264 PMC10527041

[19] Wu MM, Wang QM, Huang BY, et al. Dioscin ameliorates murine ulcerative colitis by regulating macrophage polarization. Pharmacol Res. 2021;172:105796. doi: 10.1016/j.phrs.2021.105796 34343656

[20] Guo X, Fereydooni A, Isaji T, et al. Inhibition of the Akt1-mTORC1 axis alters venous remodeling to improve arteriovenous fistula patency. Sci Rep. 2019;9(1):11046. doi: 10.1038/s41598-019-47542-5 31363142 PMC6667481

[21] Kimura T, Nada S, Takegahara N, et al. Polarization of M2 macrophages requires Lamtor1 that integrates cytokine and amino-acid signals. Nat Commun. 2016;7(1):13130. doi: 10.1038/ncomms13130 27731330 PMC5064021

[22] Collins SL, Oh MH, Sun IH, et al. mTORC1 signaling regulates proinflammatory macrophage function and metabolism. J Iimmunol (Baltim, Md : 1950). 2021;207(3):913–922. doi: 10.4049/jimmunol.2100230 34290107

[23] Hallowell RW, Collins SL, Craig JM, et al. mTORC2 signalling regulates M2 macrophage differentiation in response to helminth infection and adaptive thermogenesis. Nat Commun. 2017;8(1):14208. doi: 10.1038/ncomms14208 28128208 PMC5290163

[24] Ouyang X, Han Y, Qu G, et al. Metabolic regulation of T cell development by Sin1-mTORC2 is mediated by pyruvate kinase M2. J Mol Cell Biol. 2019;11(2):93–106. doi: 10.1093/jmcb/mjy065 30428057 PMC6392101

[25] Wu L, Zhang X, Zheng L, et al. RIPK3 orchestrates fatty acid metabolism in tumor-associated macrophages and hepatocarcinogenesis. Cancer Immunol Res. 2020;8(5):710–721. doi: 10.1158/2326-6066.CIR-19-0261 32122992

[26] Liu J, Wang C. Lysophosphatidic acid is associated with oocyte maturation by enhancing autophagy via PI3K-AKT-mTOR signaling pathway in granulosa cells. J Ovarian Res. 2023;16(1):137. doi: 10.1186/s13048-023-01228-9 37434211 PMC10334515

[27] Jacqueline C, Dracz M, Boothman S, et al. Identification of cell surface molecules that determine the macrophage activation threshold associated with an early stage of malignant transformation. Front Immunol. 2021;12:749597. doi: 10.3389/fimmu.2021.749597 34712237 PMC8546176

[28] Zhang L, Li Y, Wang Y, et al. mTORC2 facilitates liver regeneration through sphingolipid-induced PPAR-α-fatty acid oxidation. Cell Mol Gastroenterol Hepatol. 2022;14(6):1311–1331. doi: 10.1016/j.jcmgh.2022.07.011 35931382 PMC9703135

[29] Lu F, Ye M, Shen Y, et al. Hypoxic tumor-derived exosomal miR-4488 induces macrophage M2 polarization to promote liver metastasis of pancreatic neuroendocrine neoplasm through RTN3/FABP5 mediated fatty acid oxidation. Int J Biol Sci. 2024;20(8):3201–3218. doi: 10.7150/ijbs.96831 38904015 PMC11186367

[30] Guo Z, Zhao K, Feng X, et al. mTORC2 regulates lipogenic gene expression through PPARγ to control lipid synthesis in bovine mammary epithelial cells. Biomed Res Int. 2019;2019:1–11. doi: 10.1155/2019/5196028 PMC6541957 31223619

[31] Hu W, Jiang C, Kim M, et al. Isoform-specific functions of PPARγ in gene regulation and metabolism. Genes Dev. 2022;36(5–6):300–312. doi: 10.1101/gad.349232.121 35273075 PMC8973844

[32] Hong Y, Jiang L, Tang F, et al. PPAR-γ promotes the polarization of rat retinal microglia to M2 phenotype by regulating the expression of CD200-CD200R1 under hypoxia. Mol Biol Rep. 2023;50(12):10277–10285. doi: 10.1007/s11033-023-08815-5 37971567

[33] Liu S, Zhang H, Li Y, et al. S100A4 enhances protumor macrophage polarization by control of PPAR-γ-dependent induction of fatty acid oxidation. J Immunother Cancer. 2021;9(6):e002548. doi: 10.1136/jitc-2021-002548 34145030 PMC8215236

[34] Su P, Li O, Ke K, et al. Targeting tumor‑associated macrophages: critical players in tumor progression and therapeutic strategies (review). Int J Oncol. 2024;64(6). doi: 10.3892/ijo.2024.5648 PMC11087038 38695252

[35] Minopoli M, Sarno S, Cannella L, et al. Crosstalk between macrophages and myxoid liposarcoma cells increases spreading and invasiveness of tumor cells. Cancers (Basel). 2021;13(13):3298. doi: 10.3390/cancers13133298 34209309 PMC8268435

[36] Fedor A, Bryniarski K, Nazimek K. mTOR signaling in macrophages: all depends on the context. Int J Mol Sci. 2025;26(15):7598. doi: 10.3390/ijms26157598 40806725 PMC12347599

[37] Haloul M, Oliveira ERA, Kader M, et al. mTORC1-mediated polarization of M1 macrophages and their accumulation in the liver correlate with immunopathology in fatal ehrlichiosis. Sci Rep. 2019;9(1):14050. doi: 10.1038/s41598-019-50320-y 31575880 PMC6773708

[38] Liu L, Qin Y, Cai Z, et al. Corrigendum to: effective-components combination improves airway remodeling in COPD rats by suppressing M2 macrophage polarization via the inhibition of mTORC2 activity. Phytomedicine. 2022;98:153976. doi: 10.1016/j.phymed.2022.153976 35158235

[39] O’Brien SL, Tripp E, Barki N, et al. Intracrine FFA4 signaling controls lipolysis at lipid droplets. Nat Chem Biol. 2025;22(1):109–119. doi: 10.1038/s41589-025-01982-5 40764678 PMC12727528

